# Supplementary material for: The genome sequence of the Bordered Sallow moth, Pyrrhia umbra (Hufnagel, 1766)
Source: Wellcome Open Res. 2025 Apr 23;10:208. [Version 1] doi: 10.12688/wellcomeopenres.24001.1 (PMC12411841; doi:10.12688/wellcomeopenres.24001.1)
Supplement: Peer review history

# Reviewer report: Reviewer response for version 1

**DOI:** 10.21956/wellcomeopenres.26479.r129100

**Published:** 2025-09-04

**Authors:** Doga Cedden

**Affiliations:** 1 University of Göttingen, Göttingen, Germany

The data note reports a genome assembly from the lepidopteran *Pyrrhia umbra. *The author provides sufficient quality control of the assembly with adequately described methodology. The genome assembly is of high quality with a BUSCO score of 98.9% and a *k*-mer recovery of 99.62%. The raw data and the assembly are accessible from the provided accession. I have no further comments.

Are sufficient details of methods and materials provided to allow replication by others?

Yes

Is the rationale for creating the dataset(s) clearly described?

Yes

Are the datasets clearly presented in a useable and accessible format?

Yes

Are the protocols appropriate and is the work technically sound?

Yes

Reviewer Expertise:

I work on coleopteran pests using RNAi and RNA-seq.

I confirm that I have read this submission and believe that I have an appropriate level of expertise to confirm that it is of an acceptable scientific standard.

# Reviewer report: Reviewer response for version 1

**DOI:** 10.21956/wellcomeopenres.26479.r123515

**Published:** 2025-06-12

**Authors:** Wei JIufeng

**Affiliations:** 1 Shanxi Agricultural University, Taigu, China

The manuscript title “The genome sequence of the Bordered Sallow moth, *Pyrrhia umbra *(Hufnagel, 1766)”sequence has a total length of 433.58 megabases. The data and methodology are credible, and the results are substantial. We recommend the manuscript for indexing publication after address some minor question.

 Comment 1: key words: Species names should be italicized;

 Comment 2: Can the species photographs be clearer?

Are sufficient details of methods and materials provided to allow replication by others?

Yes

Is the rationale for creating the dataset(s) clearly described?

Yes

Are the datasets clearly presented in a useable and accessible format?

Yes

Are the protocols appropriate and is the work technically sound?

Yes

Reviewer Expertise:

Genome Evolution and Phylogenetic Study of Scale Insects (Coccoidea)

I confirm that I have read this submission and believe that I have an appropriate level of expertise to confirm that it is of an acceptable scientific standard.
